# Grasping time does not influence the early adherence of aperture shaping to Weber's law

**DOI:** 10.3389/fnhum.2012.00332

**Published:** 2012-12-21

**Authors:** Matthew Heath, Scott A. Holmes, Ali Mulla, Gordon Binsted

**Affiliations:** ^1^NeuroBehavioural Lab, School of Kinesiology and Graduate Program in Neuroscience, University of Western OntarioLondon, ON, Canada; ^2^Sensorimotor Neuroscience Lab, Faculty of Health and Social Development, University of British ColumbiaKelowna, BC, Canada

**Keywords:** action, grasping, impulse-variability, psychophysical, Weber's law

## Abstract

The “just noticeable difference” (JND) represents the minimum amount by which a stimulus must change to produce a noticeable variation in one's perceptual experience (i.e., Weber's law). Recent work has shown that within-participant standard deviations of grip aperture (i.e., JNDs) increase linearly with increasing object size during the early, but not the late, stages of goal-directed grasping. A visually based explanation for this finding is that the early and late stages of grasping are respectively mediated by relative and absolute visual information and therefore render a time-dependent adherence to Weber's law. Alternatively, a motor-based explanation contends that the larger aperture shaping impulses required for larger objects gives rise to a stochastic increase in the variability of motor output (i.e., impulse-variability hypothesis). To test the second explanation, we had participants grasp differently sized objects in grasping time criteria of 400 and 800 ms. Thus, the 400 ms condition required larger aperture shaping impulses than the 800 ms condition. In line with previous work, JNDs during early aperture shaping (i.e., at the time of peak aperture acceleration and peak aperture velocity) for both the 400 and 800 ms conditions scaled linearly with object size, whereas JNDs later in the response (i.e., at the time of peak grip aperture) did not. Moreover, the 400 and 800 ms conditions produced comparable slopes relating JNDs to object size. In other words, larger aperture shaping impulses did not give rise to a stochastic increase in aperture variability at each object size. As such, the theoretical tenets of the impulse-variability hypothesis do not provide a viable framework for the time-dependent scaling of JNDs to object size. Instead, we propose that a dynamic interplay between relative and absolute visual information gives rise to grasp trajectories that exhibit an early adherence and late violation to Weber's law.

## Introduction

Our ability to distinguish the perceptual features of a visual target is mediated by relative visual information. For example, judging the size of the cup containing our morning coffee is determined by the size of other objects within the visual scene as well as prior experiences with the cup. In contrast, reaching to grasp the cup requires that the visuomotor system access absolute (i.e., Euclidean) visual information to successfully grasp it. Notably, however, an issue of continued debate is whether grasping trajectories are restrictively mediated via absolute visual information or exhibit a time-dependent use of relative and absolute visual information. On the one hand, some studies from the pictorial illusions literature have shown that grip aperture shaping is refractory to the context-dependent properties (i.e., relative visual information) of pictorial illusions (e.g., Ebbinghaus illusion, Müller-Lyer figures: Danckert et al., 2002; Heath et al., 2005). Such a result is consistent with the perception/action model's (PAM: Goodale and Milner, 1992) assertion that unitary and absolute visual information mediates aperture shaping (for recent review see Goodale, 2011). Indeed, the PAM states that relative visual information is used only for perceptual judgments or a motor response implemented following a period of visual occlusion (e.g., Hu and Goodale, 2000; Westwood and Goodale, 2003). On the other hand, Glover and Dixon (2001) have reported that pictorial illusions influence the early, but not late, stages of grip aperture shaping. This “dynamic illusion effect” laid the foundation for Glover's (2004) planning/control model (PCM) and the contention that the early and late stages of action are mediated by relative and absolute visual information, respectively.

It is, however, important to note that the extent to which grip aperture is “tricked” by a pictorial illusion is dependent on methodological factors such as when and what measure is used to assess motor output (e.g., Franz, 2001; Glover, 2004) and whether the genesis of the illusion arises from early (i.e., striate cortex) or later (i.e., inferotemporal cortex) visual processing structures (Dyde and Milner, 2002). Furthermore, intrinsic and extrinsic properties such as attentional demands, practice, and the implementation of distinct movement strategies have been shown to influence the visuomotor system's sensitivity to pictorial illusions (Mendoza et al., 2005; Gonzalez et al., 2006; Heath et al., 2006; Bruno et al., 2008; Neely et al., 2008).

In redress to the limitations of pictorial illusions, Ganel et al. (2008) had participants grasp differently sized objects (20, 30, 40, 50, 60, and 70 mm) placed within a neutral visual background and applied the psychophysical principles of Weber's law to examine the nature of the visual information mediating grip aperture. In particular, Weber's law states that changes in a stimulus that will be “just noticeable” is a constant ratio of the original stimulus magnitude and that the sensitivity of detecting a change in any physical continuum is *relative* as opposed to absolute. Thus, and as stated by Ganel et al., the just noticeable difference (JND) for weaker stimuli is smaller and the resolution is greater than more robust stimuli in the same sensory continuum. In Ganel et al.'s study, within-participant standard deviations of grip aperture (i.e., the JNDs) were computed during manual estimation (i.e., perceptual task) and grasping (i.e., motor task) tasks to determine participants' sensitivity to detecting changes in object size. In terms of the perceptual task, JNDs increased in relation to increasing object size; that is, the trial-to-trial stability of participants estimation of the size difference between their grip aperture (i.e., the comparator stimulus) and the target object decreased as a function of increasing stimulus intensity (i.e., the object size). In contrast, the motor task elicited a null relationship between JNDs and object size. Thus, perceptual and motor tasks elicited a fundamental adherence and violation of Weber's law, respectively. Ganel et al. interpreted their results within the PAM's framework that relative visual information supports perceptions and absolute visual information supports actions.

**Figure 1:**
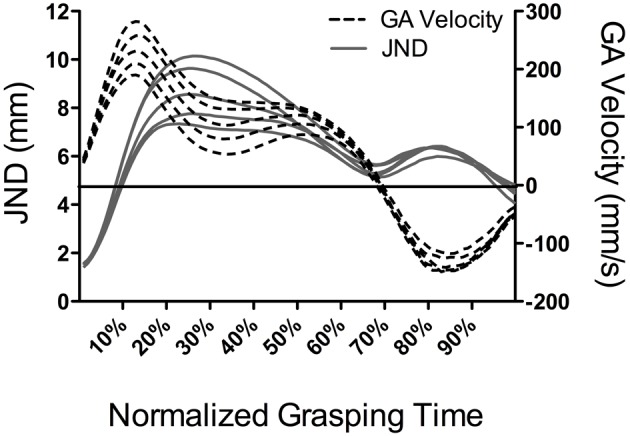
**Results from Holmes et al. (2011) showing JND magnitudes (mm: solid lines and left ordinate) and grip aperture (GA) velocities (mm/s: dotted lines and right ordinate) as function of object size (20, 30, 40, 50, and 60 mm) at percentile increments of normalized grasping time.** The horizontal line represents the zero crossing for aperture velocity. For ease of presentation, we did not include different symbols or line weightings to distinguish the different object sizes. However, the figure clearly depicts an increase in JNDs and GA velocities with increasing object size from approximately 10 through 50% of grasping time. During the later stages of aperture shaping, JNDs, and GA velocities do not reliably scale to object size.

A notable feature of Ganel et al.'s (2008) work was that JNDs in the motor task were computed at the time of peak grip aperture. Indeed, because peak grip aperture is a late occurring metric (~75% of grasping time: Jeannerod, 1984) it is unclear from their work whether JNDs elicit a time-independent or time-dependent violation of Weber's law. To address that limitation, Heath and colleagues (Heath et al., 2011; Holmes et al., 2011) employed the same methods of Ganel et al.; however, JNDs were computed at decile increments of grasping time (i.e., 10, 20, … 80, and 90%) as well as the time of peak grip aperture. In addition, responses were completed in conditions wherein vision was continuously available to participants and when vision was occluded at, or for some period of time prior to, movement onset. Results showed that JNDs during early aperture shaping increased linearly as a function of increasing object size whereas JNDs later in the response (i.e., >50% of grasping time), and including the time of peak grip aperture, did not: a result that was consistent across visual conditions. One interpretation for these findings can be drawn from the PCM's contention that relative and absolute visual information respectively mediate the early and late stages of grip aperture shaping. In other words, the early adherence and late violation of grasping to Weber's law reflects the visual properties of the to-be-grasped object. An alternative explanation, however, may be drawn from the impulses (i.e., force over time) involved in aperture shaping and their extant influence on motor output variability (i.e., impulse-variability hypothesis: Schmidt et al., 1979). Indeed, Schmidt et al. (1979) demonstrated a linear relationship between the amount of force produced and the within-participant variability of that force production (i.e., the JNDs in this study) for isometric “shots” of force as well as goal-directed reaches (see also Sherwood and Schmidt, 1980; Carlton and Newell, 1993). As such, the larger impulses required for the rapid and early scaling of grip aperture for objects of increasing size may give rise to increased JNDs. In demonstration, Figure 1 presents data from an earlier study by Holmes et al. (2011) showing JND magnitudes and grip aperture velocities for differently sized target objects at percentile increments of grasping time. Notably, grasping times in this study were roughly constant across the different object sizes. As such, Figure 1 shows that grip aperture velocities increased in relation to increasing object size early in the response and that the timeline of this scaling was commensurate with target-dependent changes between JNDs and object size. This observation suggests that the early adherence of grip aperture to Weber' s law may not relate to the visual properties of the target object *per se*; rather, it may reflect stochastic properties associated with impulse-variability during early aperture shaping.

**Figure 2:**
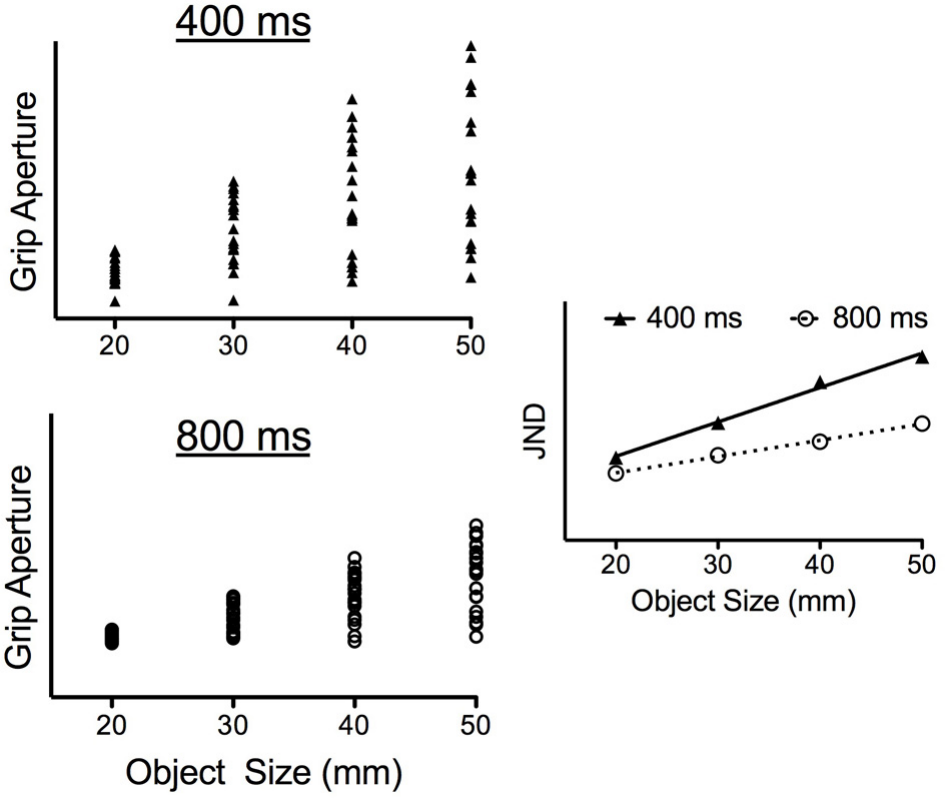
**Theoretical data demonstrating impulse-variability predictions for trial-to-trial changes in grip aperture (i.e., the JNDs) as a function of object size and grasping time condition.** In particular, the left panels of this figure present grip aperture size for a theoretical participant at some early point in aperture shaping (e.g., peak aperture acceleration) across 20 trials to each of four object sizes in both the 400 ms (left top panel) and 800 ms (left bottom panel) conditions. For both conditions, impulse-variability predicts a stochastic increase in the range of trial-to-trial grip apertures with increasing object size (i.e., JNDs increase linearly with increasing object size). Additionally, the shortened grasping time in the 400 ms condition requires larger aperture opening impulses and is therefore predicted to result in an increased range of grip apertures for each object size in comparison to matched objects in the 800 ms condition. As shown in the right panel, this between-condition difference would render a steeper slope relating JNDs to object size in the 400 ms as compared to the 800 ms condition. In particular, this figure demonstrates a twofold between-condition difference in the JND/object size slopes.

The present study manipulated the speed of grasping responses to determine whether impulse-variability accounts for the early scaling of JNDs to object size. To accomplish that objective, participants grasped differently sized objects (20, 30, 40, and 50 mm) placed at a common location in conditions wherein responses were completed in 400 and 800 ms. The 400 ms criterion was selected based on pilot work showing it to represent the shortest time in which participants were able to successfully grasp the target objects used here. In turn, the 800 ms condition was selected based on previous work showing this time frame to represent the speed of self-paced grasping (Heath et al., 2011; Holmes et al., 2011). Most notably, and because a common target location was used here, the 400 ms condition required an increased rate (i.e., acceleration) and speed (i.e., velocity) of aperture opening and closing (i.e., larger movement impulses) to successfully grasp the target object. Thus, if impulse-variability gives rise to the time-dependent scaling of JNDs to object size, then the slopes relating JNDs to object size during early aperture shaping (e.g., peak aperture acceleration) should be steeper in the 400 as compared to 800 ms condition. To underscore this prediction, the left panels of Figure 2 present theoretical trial-to-trial grip aperture values at some early point in a response for each of four objects (i.e., 20, 30, 40, and 50 mm) separately for 400 and 800 ms grasping time conditions^1^. As predicted by the impulse-variability theory, Figure 2 shows that the larger impulses required to grasp progressively larger objects produce a stochastic increase in the trial-to-trial variability of grip aperture (i.e., the JNDs) in both the 400 and 800 ms condition. Additionally, and because the impulses required for a successful grasp must be increased when grasping time is shortened, the right panel of Figure 2 shows that the slopes relating JNDs to object size are steeper in the 400 ms as compared to 800 ms condition. In contrast, if the visual representation of object size subserves the early adherence of grip aperture to Weber's law, then JND/object size scaling should be comparable across the different grasping time conditions. In other words, results would support the PCM's contention that relative visual information supports early aperture shaping.

## Materials and methods

### Participants

Fifteen (11 male and 4 female: age range = 20–28 years of age) self-declared right-hand dominant individuals with normal, or corrected-to-normal, vision were recruited for this study. Participants provided written informed consent and this work was approved by the Office of Research Ethics, University of Western Ontario, and conducted in accordance with the Declaration of Helsinki.

### Apparatus and stimuli

Participants stood in front of a table (height of 880 mm: surface width and depth of 1040 and 740 mm, respectively) for the duration of the experiment. Target objects were acrylic blocks painted flat black and were 20, 30, 40, and 50 mm in length and 10 mm in depth and height and were presented against a flat white surface (i.e., neutral visual background). For all trials, the long-axis of target objects was oriented perpendicular to the midline of participants and was located 500 mm from the front edge of the tabletop (i.e., grasping movements were completed in the depth plane). A pressure sensitive switch secured to the tabletop midline, and located 50 mm from its front edge, served as the start location for each trial. Vision of the grasping environment was manipulated via liquid-crystal occlusion goggles (PLATO Translucent Technologies, Toronto, ON, Canada) and MATLAB (7.6: The Mathworks, Natick, MA, USA) and the Psychophysics Toolbox extensions (ver 3.0; see Brainard, 1997) were used to control visual and auditory events.

### Procedures

Prior to each trial, the occlusion goggles were set to their translucent state while the experimenter placed the appropriate target object on the tabletop. During this time, participants rested their grasping (i.e., right) hand on the start location with their thumb and forefinger pinched lightly together. Following placement of the appropriate target object, the occlusion goggles were set to their transparent state for a visual preview (randomized between any value from 2000 to 3000 ms). The randomized preview was used to prevent participants from anticipating response cuing. After the preview phase, an auditory tone cued participants to grasp the long-axis of the target object with their thumb and forefinger (i.e., precision grip) in each of the two grasping time conditions (see below). At movement onset (i.e., release of pressure from the start location) the occlusion goggles reverted to their translucent state thereby providing an open-loop grasping environment. Participants were instructed to maintain their grasp, but not lift, the target object until prompted by the experimenter to move back to the start location.

The two conditions used here entailed grasping time criterion of 400 and 800 ms and were performed in separate and counterbalanced blocks entailing 80 trials each. Prior to data acquisition in each block, participants completed a number of practice trials such that five successive trials were completed within a bandwidth (±50 ms) about the required grasping time criterion. Following each practice trial, participants were provided verbal knowledge of results describing their performance: a grasping time more than 50 ms below or above the required criterion was described as “too fast” or “too slow,” respectively, whereas a grasping time within 50 ms of the criterion was described as “good.” On average, 12 (SD = 8) practice trials were required. Following the practice trials, participants completed 20 acquisition trials to each of the four target objects. The presentation of object size within each block was randomized. As per the methods above, knowledge of results regarding grasping time performance continued during acquisition trials.

### Data analysis

Movement of the grasping limb was tracked via infrared emitting diodes (IREDs) placed on the styloid process of the wrist, the medial aspect of the distal phalanx of the thumb and the lateral surface of the distal phalanx of the forefinger. Displacement of the IREDs was sampled at 400 Hz via an Optotrak Certus (Northern Digital Inc. Waterloo, ON, Canada). Offline displacement data were filtered via a second-order dual-pass Butterworth filter employing a low-pass cut-off frequency of 15 Hz. Instantaneous velocities were computed from displacement data via a five-point central finite difference algorithm. In turn, instantaneous accelerations were computed from velocity via the same algorithm. Movement onset was marked by release of pressure from the start position switch and movement offset was marked as the first frame wherein resultant wrist velocity fell below a value of 50 mm/s for 20 consecutive frames (i.e., 50 ms).

### Dependent variables and statistical analyses

We examined results for grasping time (time from movement onset to movement offset), the magnitude and percent time of peak grip aperture acceleration (peak GA_A_: maximum resultant acceleration of grip aperture opening) and peak grip aperture velocity (peak GA_V_: maximum resultant velocity of grip aperture opening) as well as grip aperture size (i.e., resultant distance between thumb and forefinger) and associated JNDs at the time of the aforementioned kinematic markers (i.e., peak GA_A_ and peak GA_V_). In addition, we computed peak grip aperture (i.e., PGA: maximum resultant distance between thumb and forefinger) and associated JNDs as well as the percent time to PGA. The aforementioned variables were examined via 2 (grasping time condition: 400 and 800 ms) by 4 (object size: 20, 30, 40, and 50 mm) repeated measures ANOVA. Significant main effects/interactions were decomposed via power-polynomials (Pedhazur, 1997).

It should be noted that a traditional “just-noticeable-difference” (JND) measures the smallest difference between an original and comparator stimulus that can be perceptually identified. For example, when presented with two separate lines the performer would be required to verbally identify which of the two lines is longer. JNDs in this situation are therefore defined statistically with correct identification dependent on an arbitrary criterion; that is, some studies may employ a 75% correct criterion for identification of the stronger stimulus, whereas other studies may employ an 85% correct criterion (or any other possible value). Notably, a statistical criterion for correct stimulus identification is not available in a grasping task. Instead, the JNDs used in this and other grasping studies (e.g., Ganel et al., 2008; Heath et al., 2011; Holmes et al., 2011) represent the within-participant standard deviations of grip aperture. According to Ganel et al., the basis for this technique is drawn from the classic method of adjustment in which variance provides a measure of visuomotor uncertainty “… for which the observer is unable to tell the difference between the size of the comparison and the target object” (p. 600). Such an approach supports Fechnerian principles of Weber functions (see Marks and Algom, 1998), and we interpret linear scaling of JNDs to increasing object size (i.e., the Weber function) as adherence to the psychophysical properties of Weber's law.

## Results

**Figure 3:**
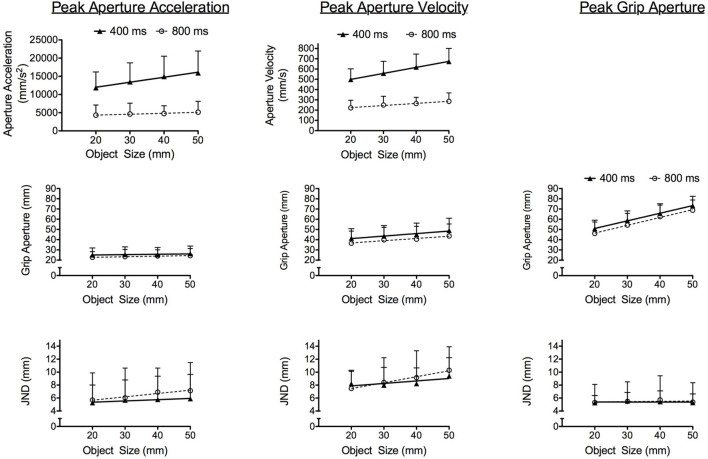
**The panels depict linear regressions of group means related to the kinematic variables analyzed at the time of peak aperture acceleration, peak aperture velocity, and peak grip aperture as a function of object size in the 400 (closed symbols and solid line) and 800 ms (open symbol and dotted line) conditions.** The left column depicts peak grip aperture acceleration (mm/s^2^) and time-matched aperture size (mm) and JNDs (mm). The middle column depicts grip aperture velocity (mm/s) and time-matched aperture size (mm) and JNDs (mm). The right column depicts peak grip aperture (mm) and associated JNDs. A panel is not included at the top right because aperture acceleration and velocity are (by definition) equal to zero at the time of peak grip aperture. Error bars represent between-participant standard deviations.

Grasping times in the 400 ms condition (424 ms SD = 26) were shorter than the 800 ms condition (795 ms SD = 29), *F*s_(1, 14)_ = 1192.00, *p*s < 0.001. The top panels of Figure 3 present results for early aperture shaping kinematics and show that peak GA_A_ and peak GA_V_ in the 400 ms condition were larger than in the 800 ms condition, *F*s_(1, 14)_ = 127.12 and 313.05, respectively, for peak GA_A_ and GA_V_, *p*s < 0.001. In addition, peak GA_A_ and peak GA_V_ elicited main effects of object size, *F*s_(3, 42)_ = 28.86 and 101.23, respectively, for peak GA_A_ and GA_V_, *p*s < 0.001, and grasping time condition by object size interactions, *F*s_(3, 42)_ = 12.75 and 16.29, respectively, for peak GA_A_ and peak GA_V_, *p*s < 0.001. Peak GA_A_ and peak GA_V_ in the 400 [only linear effects significant: *F*s_(1, 14)_ = 23.67 and 101.85, respectively, for peak GA_A_ and peak GA_V_, *p*s < 0.001] and 800 ms [only linear effects significant: *F*s_(1, 14)_ = 26.01 and 115.86, respectively, for peak GA_A_ and peak GA_V_, *p*s < 0.001] conditions increased linearly as a function of increasing object size. Notably, however, Figure 3 shows that the slopes relating peak GA_A_ to object size [*t*_(14)_ = 4.19, *p* < 0.01] and peak GA_V_ to object size [*t*_(14)_ = 5.10, *p* < 0.001] were steeper in the 400 (peak GA_A_: *b* = 130 mm/s^2^ SD = 87; peak GA_V_: *b* = 6 mm/s SD = 2) than the 800 ms (peak GA_A_: *b* = 23 mm/s^2^ SD = 20; peak GA_V_: *b* = 2 mm/s SD = 1) condition. Results for the percent time to peak GA_A_ and peak GA_V_ showed that the timing of each did not vary across the 400 (peak GA_A_: 20% SD = 6, peak GA_V_: 31% SD = 8) and 800 ms (peak GA_A_: 19% SD = 11, peak GA_V_: 32% SD = 16) conditions (*F* < 1.2)^2^ (Table 1). In terms of the timing of our later occurring grasping kinematic, the percent time to PGA elicited main effects for grasping time, *F*s_(1, 14)_ = 179.68, *p*s < 0.001, such that the onset of PGA occurred earlier in the 400 (61% SD = 7) as compared to the 800 ms (75% SD = 13) condition.

**Table 1:** **Percent time to peak aperture acceleration, peak aperture velocity, and peak grip aperture in the 400 and 800 ms conditions and as a function of object size (20, 30, 40, and 50 mm)**.

	**400 ms**				**800 ms**			
	**20 mm**	**30 mm**	**40 mm**	**50 mm**	**20 mm**	**30 mm**	**40 mm**	**50 mm**
%TPA_A_	23 (8)	22 (8)	21 (7)	20 (6)	19 (12)	19 (10)	18 (10)	18 (10)
%TPA_V_	33 (9)	32 (9)	30 (8)	30 (8)	33 (18)	33 (16)	31 (15)	32 (15)
%TPGA	60 (4)	61 (4)	61 (4)	61 (4)	75 (4)	75 (4)	76 (4)	77 (4)

*Values in parentheses represent between-participant standard deviations*.

*Note: %TPA_A_, percent time to peak aperture acceleration; %TPA_V_, percent time to peak aperture velocity; and %TPGA, percent time to peak grip aperture*.

**Figure 4:**
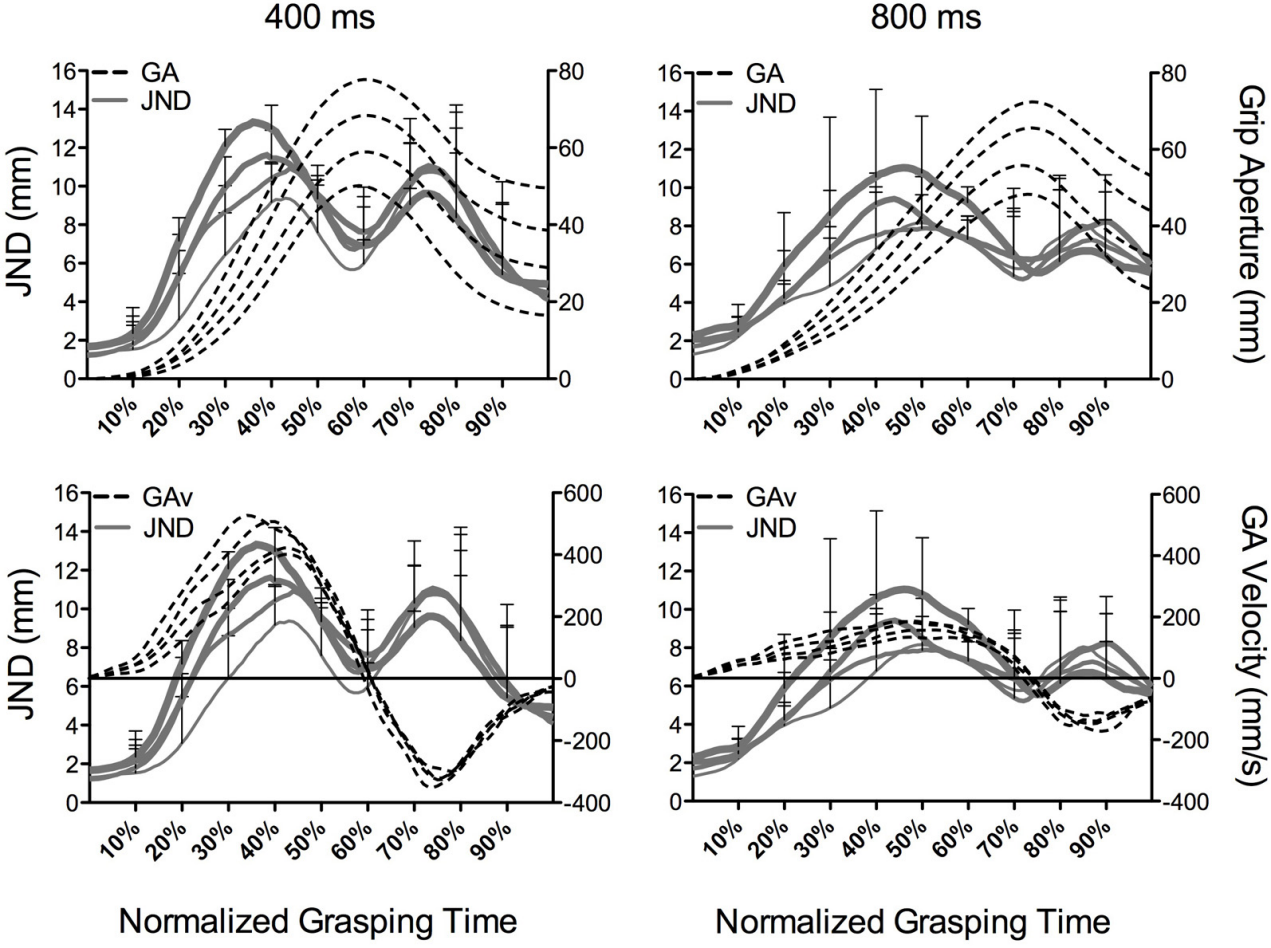
**The top panels represent group JNDs (mm: solid lines and left ordinate) and grip aperture (GA) size (mm: dotted lines and right ordinate) and the bottom panels depict JNDs and grip aperture velocities (mm/s: dotted lines and right ordinate) as a function of object size (20, 30, 40, and 50 mm) at percentile increments of grasping time in the 400 and 800 ms conditions.** The horizontal line in the bottom panels represent the zero crossing for GA velocity and the first zero crossing during the later stages of the trajectory denotes the time to peak grip aperture. The increased line weighting for JNDs in this figure correspond to an increase in object size. JND error bars are presented at the deciles and represent between-participant standard deviations.

Before turning to our quantitative examination of grip aperture size and associated JNDs, we provide a qualitative description of time normalized aperture trajectories. In line with earlier work (e.g., Heath et al., 2011; Holmes et al., 2011), Figure 4 presents grip aperture size and associated JNDs (top panels) as well as grip aperture velocity and JNDs (bottom panels) for the different object sizes at percentile increments of grasping time in the 400 and 800 ms conditions. A number of salient features can be gleaned from this figure. First, grip aperture in the 400 and 800 ms conditions demonstrated an early (~10% of grasping time) and continuous scaling to object size with peak grip aperture occurring earlier in the 400 as compared to the 800 ms condition. Second, aperture velocities in the opening phase of the 400 ms condition were larger than the 800 ms condition. Third, JNDs in the 400 and 800 ms conditions produced a time-dependent scaling to object size; that is, JNDs increased with increasing object size early in the grasp trajectory and up to a point prior to peak grip aperture. Fourth, the time-dependent scaling of JNDs to object size roughly corresponded to that associated with the scaling of grip aperture velocity to object size. Fifth, Figure 4 shows that the magnitude of JND/object size scaling is comparable across the 400 and 800 ms conditions. Further, partial correlations relating GA_V_ and JNDs at deciles (i.e., 10–90%) of grasping time were computed by removing the linear relations of object size. Correlation coefficients, computed separately for the 400 and 800 ms conditions, produced reliable relationships at each decile (*p* < 0.01). Additionally, Fisher r-to-z transformations (Snedecor and Cochran, 1980) of coefficients at time-matched values of grasping time did not yield reliable between-condition differences (*p* = ns). Thus, GA_V_ and JND values are reliably related throughout aperture shaping and this relationship is *not* driven by the effect of object size. More notably, the absence of between-condition differences in correlation coefficients indicates that the different grasping time conditions used here did not differentially influence the magnitude of the linear relations between GA_V_ and JND values.

In terms of our quantitative examination, we computed grip aperture size and associated JNDs at the absolute time of peak GA_A_, peak GA_V_, and PGA. This approach avoids the potential pitfalls associated with time normalizing trajectories with different grasping times and therefore provides a basis for determining whether the absolute rate and speed of aperture opening influenced JND/object size scaling. Concerning the early kinematic markers, grip aperture values at the time of peak GA_A_ and peak GA_V_ produced main effects for grasping time condition, *F*s_(1, 14)_ = 4.66 and 5.57, respectively, for peak GA_A_ and peak GA_V_, *p*s < 0.05, and object size, *F*s_(3, 42)_ = 5.25 and 45.48, respectively, for peak GA_A_ and peak GA_V_, *p*s < 0.01. The middle panels of Figure 3 show that grip aperture values at the time of peak GA_A_ and peak GA_V_ were larger in the 400 as compared to 800 ms condition, and in both conditions grip aperture increased linearly with increasing object size [only linear effects significant: *F*s_(1, 14)_ = 10.15 and 93.65, respectively, for peak GA_A_ and peak GA_V_, *p*s < 0.01]. Results for JNDs at the time of peak GA_A_ and peak GA_V_ produced significant main effects for object size, *F*s_(3, 42)_ = 4.02 and 8.11, respectively, for peak GA_A_ and peak GA_V_, *p*s < 0.001, such that values increased linearly with increasing object size [only linear effects significant: *F*s_(1, 14)_ = 4.48 and 13.28, respectively, for peak GA_A_ and peak GA_V_, *p*s < 0.05]. Notably, however, both peak GA_A_ and peak GA_V_ demonstrated null grasping time condition by object size interactions (*F* < 1.2). As such, the scaling of JNDs to object size did not vary across the 400 and 800 ms conditions. Concerning our later occurring kinematic marker, results for PGA indicated main effects for grasping time condition, *F*s_(1, 14)_ = 7.07, *p*s < 0.02, and object size, *F*s_(3, 42)_ = 326.71, *p*s < 0.001. Figure 3 (see middle right panel) shows that PGA in the 400 ms condition was larger than the 800 ms condition, and in both conditions PGA increased linearly as a function of increasing object size [only linear effect significant: *F*s_(1, 14)_ = 419.13, *p*s < 0.001]. In terms of the JNDs at PGA, no significant main effects or interactions were observed (*F* < 1) (see Figure 3).

## Discussion

The present study examined whether the time-dependent scaling of JNDs to object size reflects the use of distinct visual metrics or the stochastic properties of impulse-variability in aperture shaping. To that end, we manipulated the rate and speed of aperture shaping by having participants grasp differently sized objects—at a common location—in grasping time criteria of 400 and 800 ms.

### Grasping time influences early and late aperture kinematics

Before addressing our primary research question, we outline the general impact of our grasping time manipulation on aperture shaping. Figure 3 shows that peak GA_A_ and peak GA_V_ and their associated grip apertures were larger in the 400 ms than the 800 ms condition. Figure 3 also shows that our grasping time manipulation influenced later aperture shaping such that PGA was larger in the 400 ms than the 800 ms condition. Of course, the larger GA_A_ and GA_V_ values in the 400 ms conditions are expected and indicate that the rate and speed of aperture opening was increased to ensure a successful grasp. Put another way, the temporal demands of the 400 ms condition required larger impulses for hand opening (and closing). In terms of grip aperture size, our results support Wallace and Weeks' (1988) and Wing et al.'s (1986) work showing that shorter grasping times engender larger PGAs and serve as an “error-compensating adjustment” to account for: (1) larger hand/object contact forces and, (2) increased uncertainty about the accuracy of hand/object interactions (see also Jakobson and Goodale, 1991; Bootsma et al., 1994). Moreover, as previous work has restrictively examined the impact of grasping time on PGA, the present results add importantly to the grasping literature insomuch as they demonstrate that the increase in aperture size is reflected both early (i.e., peak GA_A_ and peak GA_V_) and late (i.e., PGA) in the response. In other words, we demonstrate that the compensatory increase in grip aperture is predictive and is specified in advance of movement onset via central planning mechanisms (e.g., Arbib, 1985; Marteniuk et al., 1990).

In terms of the timing of aperture shaping, the percent times to peak GA_A_ and peak GA_V_ did not vary across the 400 and 800 ms conditions. These findings suggest that early aperture shaping kinematics are schema-based and are temporally invariant (Schmidt, 1975). In turn, results for our later occurring kinematic marker (i.e., PGA) indicated an earlier onset in the 400 ms (61%) as compared to the 800 ms (75%) condition. Recall that the 800 ms condition used here was chosen based on the self-selected grasping times reported in previous work (e.g., Heath et al., 2011). As such, the results for the 800 ms condition are consistent with Jeannerod's (1984) seminal findings that PGAs for self-selected grasping occur at approximately 75% of the response^3^. It is, however, important to recognize that studies manipulating grasping time and/or the tolerance of to-be-grasped objects have shown that participants accommodate for task difficulty by achieving PGA at progressively earlier stages in the response (Wing et al., 1986; Marteniuk et al., 1987; Wallace and Weeks, 1988). For example, Wallace and Week's had participants grasp objects in 200 and 400 ms and reported PGAs that averaged 61% of grasping time: a result paralleling the 400 ms condition used here. Presumably, the earlier onset of PGA in “fast” grasping time conditions reflects participants need to spend more time during hand closure to integrate the feedback and/or feedforward commands necessary to ensure a successful response.

### Impulse-variability does not influence JND/object size scaling

Previous work has shown that early aperture shaping exhibits a linear scaling of JNDs to object size on par to tasks involving explicit perceptual judgments (i.e., classic method of adjustment and manual estimation). In contrast, JNDs during later aperture shaping do not (Ganel et al., 2008; Heath et al., 2011; Holmes et al., 2011). Holmes et al. proposed two explanations for this finding: (1) the use of relative visual information during early aperture shaping, and (2) the stochastic properties of impulse-variability during early aperture shaping. In an attempt to test the second explanation, we manipulated the forces involved in aperture opening by requiring participants to grasp differently sized objects in criteria of 400 and 800 ms. To that end, Figure 4 provides results at percentile increments of normalized grasping time and shows that the 400 and 800 ms conditions produced an early, but not late, JND/object size scaling in line with previous work (e.g., Heath et al., 2011; Holmes et al., 2011). Recall that grasping times *within* the 400 and 800 ms condition were not influenced by object size. As such, a tentative interpretation of the independent results for the 400 and 800 ms conditions is that the larger aperture shaping impulses required for grasping progressively larger objects engendered a stochastic increase in aperture variability (i.e., a linear increase in JNDs). Importantly, however, Figures 3 and 4 demonstrate that JND/object scaling did not differ *between* conditions. Thus, and in spite of the fact that the 400 ms condition was associated with larger hand opening accelerations and velocities, the slopes relating JND to object size did not differ between the 400 and 800 ms conditions.

We recognize the potential drawback of comparing time-normalized JNDs across different grasping time conditions. Accordingly, and in addition to the qualitative description of the time-normalized data presented in the above paragraph, we computed JND/object size scaling at the absolute time of the peak rate (i.e., peak GA_A_) and speed (peak GA_V_) of aperture opening as well as the time to PGA. Results for the 400 and 800 ms conditions demonstrated an early (i.e., peak GA_A_ and peak GA_V_) but not late (i.e., PGA) scaling of JNDs to object size. Most notably, the slopes relating JNDs to object size at the time of peak GA_A_ and peak GA_V_ did not vary across the 400 and 800 ms conditions. This represents a salient finding because it demonstrates that the larger impulses involved in aperture opening in the 400 ms condition did not produce a proportionally larger increase in JND magnitudes with each object size. Thus, our results counter the notion that the stochastic properties of impulse-variability contribute to the time-dependent JND/object size scaling (Schmidt et al., 1979; see also Sherwood and Schmidt, 1980; and for more recent review Carlton and Newell, 1993). After all, impulse-variability predicts that the larger impulses required in the 400 ms condition would have produced larger JNDs in comparison to matched object sizes in the 800 ms condition (see Figure 2). Instead, we propose a visual account for our results in line with the PCM's assertion that the initial kinematic parameterization of a response is mediated via relative visual information whereas later aperture shaping is mediated via absolute visual information (Glover, 2004). Certainly such an interpretation provides a basis for why early, but not late, aperture shaping adheres to the psychophysical principles of Weber's law.

As indicated above, we believe that the present findings provide convergent support for the PCM's assertion that distinct visual metrics mediate the early and late stages of grip aperture shaping. In part, the basis for our assertion is that the scaling of JNDs to object size during early aperture shaping is commensurate to tasks involving perceptual judgments (e.g., manual estimation and classic method of adjustment: see Ganel et al., 2008; Heath et al., 2011; Holmes et al., 2011). However, we recognize that there is at least one additional interpretation for our results. In particular, Smeets and Brenner's (1999) double-pointing hypothesis contends that the digits of precision grasping are under independent control with each approaching their respective contact points orthogonally (but see van de Kamp and Zaal, 2007). Notably, as the size of a to-be-grasped object increases the movement vector required for the index finger must increase relative to the thumb to ensure equivalent accuracy at the time of object contact. Further, the double-pointing hypothesis asserts that the timing of the maximum between-digit difference is influenced by object size. As such, the more orthogonal approach associated with the index finger may engender increased variability during the early, but not late, stages of aperture shaping.

## Conclusions

Our results show that the time-dependent scaling of JNDs to object size is not related to the stochastic properties of impulse-variability. As such, we propose that the initial kinematic parameterization of a response is mediated by relative visual information and therefore gives rise to an early aperture trajectory that adheres to Weber's law. Additionally, our results underscore the importance of a continuous examination of aperture shaping to better identify the dynamic interplay between the relative and absolute visual cues mediating motor output.

### Conflict of interest statement

The authors declare that the research was conducted in the absence of any commercial or financial relationships that could be construed as a potential conflict of interest.

## Footnotes

^1^This is a simplise model wherein a random distribution about a central value increased in relation to increasing object size and was such that the central value for the 800 ms condition was 80% of that associated with the 400 ms condition. Further, a monotonically smaller distribution range was set for the 800 ms as compared to 400 ms condition.

^2^By convention we do not report all non-significant effects or interactions; however, we elected to outline F-ratios for some non-significant results to demonstrate that our grasping time manipulation did not reliably modulate specific spatiotemporal features of aperture shaping. Moreover, the sizes of the reported F-ratios indicate that the null results cannot be attributed to an inadequate replication sample size (Keppel, 1991).

^3^The group mean for the percent time to peak grip aperture in Jeannerod's (1984) work (*N* = 7) was 75.7% (*SD* = 7) and individual participant means ranged from 61.4% to 81.7% (see Table 2 of Jeannerod, 1984).
